# Identification of Secreted Proteins Involved in Nonspecific dsRNA-Mediated *Lutzomyia longipalpis* LL5 Cell Antiviral Response

**DOI:** 10.3390/v10010043

**Published:** 2018-01-18

**Authors:** Andrea Martins-da-Silva, Erich Loza Telleria, Michel Batista, Fabricio Klerynton Marchini, Yara Maria Traub-Csekö, Antonio Jorge Tempone

**Affiliations:** 1 Laboratório de Biologia Molecular de Parasitas e Vetores, Instituto Oswaldo Cruz-Fiocruz, Av. Brasil 4365, Rio de Janeiro 21040-360, RJ, Brazil; amartinsdasilva@yahoo.com.br (A.M.-d.-S.); erichlt@ioc.fiocruz.br (E.L.T.); 2 Laboratório de Genômica Funcional, Instituto Carlos Chagas—Fiocruz, Rua Prof. Algacyr Munhoz Mader 3775, Curitiba 81350-010, PR, Brazil; mbatista@fiocruz.br (M.B.); fabricio.marchini@fiocruz.br (F.K.M.); 3 Plataforma Espectrometria de Massas—RPT02H, Instituto Carlos Chagas—Fiocruz, Rua Prof. Algacyr Munhoz Mader 3775, Curitiba 81350-010, PR, Brazil

**Keywords:** *Lutzomyia longipalpis*, embryonic LL5 cells, antiviral response, interferon-like response, poly (I:C), exoproteome

## Abstract

Hematophagous insects transmit infectious diseases. Sand flies are vectors of leishmaniasis, but can also transmit viruses. We have been studying immune responses of *Lutzomyia longipalpis*, the main vector of visceral leishmaniasis in the Americas. We identified a non-specific antiviral response in *L. longipalpis* LL5 embryonic cells when treated with non-specific double-stranded RNAs (dsRNAs). This response is reminiscent of interferon response in mammals. We are investigating putative effectors for this antiviral response. Secreted molecules have been implicated in immune responses, including interferon-related responses. We conducted a mass spectrometry analysis of conditioned medium from LL5 cells 24 and 48 h after dsRNA or mock treatment. We identified 304 proteins. At 24 h, 19 proteins had an abundance equal or greater than 2-fold change, while the levels of 17 proteins were reduced when compared to control cells. At the 48 h time point, these numbers were 33 and 71, respectively. The two most abundant secreted peptides at 24 h in the dsRNA-transfected group were phospholipid scramblase, an interferon-inducible protein that mediates antiviral activity, and forskolin-binding protein (FKBP), a member of the immunophilin family, which mediates the effect of immunosuppressive drugs. The transcription profile of most candidates did not follow the pattern of secreted protein abundance.

## 1. Introduction

Insect-borne diseases afflict billions of people throughout the world. These illnesses are caused by different pathogens such as helminths, protists, bacteria, and viruses, which are the etiologic agents of diseases such as filariasis, leishmaniasis, babesiosis and dengue. Among the most important insect vectors are mosquitos and sand flies [1,2]. Phlebotomine sandflies from the genera *Phlebotomus* and *Lutzomyia* are the main vectors of *Leishmania* in the Old and New World, respectively. In the Old World, these dipterans are also proven virus vectors of *Phlebovirus, Vesiculovirus* and *Orbivirus* [3,4,5]. Despite the lack of evidence proving the vectorial capacity of New World sandflies in the transmission of viruses to humans, the occurrence of diverse virus species naturally infecting *Lutzomyia* spp. populations has been demonstrated [6,7,8,9].

Among insect transmitted diseases, the arboviruses are among the most devastating infectious illnesses in our planet. Chikungunya virus (CHIKV), West Nile virus (WNV) and dengue virus (DENV) are three of a large number of human pathogenic arthropod-borne viruses [10]. Also, the world recently witnessed the reemergence of Zika virus (ZIKV), which is associated with severe human neurological pathologies in adults and fetuses [11].

The predominant strategy for controlling vector-borne diseases consists of targeting the vectors, mostly using insecticides. The deleterious side effects of insecticide usage are widely known. Among these are consequences to ecosystems, animal and human health, and development of resistance, pointing to the urgency of developing new control strategies. The understanding of biological mechanisms involved in the relationship between pathogens and vectors can provide new approaches for the development of control strategies. The infection of *Aedes aegypti* with *Wolbachia* [12] is an excellent example of a novel arbovirus control strategy. Our group has been studying the interaction and immune responses of the sand fly *Lutzomyia longipalpis* (Diptera: Psychodidae: Phlebotominae), the most important vector of American visceral leishmaniasis [13], when infected with different pathogens [14,15,16]. The establishment of continuous insect cell lines has been an important tool for the study of insect biology. Cells from *Drosophila melanogaster* (S2), *Aedes albopictus* (C6/36), and *Anopheles gambie* (Sua5B), among other insect cell lines, have been employed to scrutinize diverse features of insect immunity [17,18,19]. We have detected complex immune responses in the *Lutzomyia longipalpis* LL5 embryonic cell line [20] when challenged by different pathogens [21]. We have also identified a non-specific antiviral response in these cells when transfected with non-specific double-stranded RNA (dsRNA) [22], in a way similar to interferon response in mammals [23]. After dsRNA transfection, regardless of nucleotide sequence, there was no replication of a West Nile reporter virus that expresses luciferase and lacks structural genes, as indicated by a lack of luciferase production [24]. We are presently searching for molecules responsible for this response.

Secreted proteins have a key role in biological processes involving cell signaling. Naïve cells can be modulated by viral or host components transferred from neighboring infected cells via the release of extracellular vesicles and secretion of soluble molecules, leading to activation of host immune response and changes in the secreted protein repertoire [25,26,27]. In order to investigate the effect of polyinosinic:polycytidylic acid (poly I:C) transfection on the protein secretion profile of *L. longipalpis* LL5 cells, we carried out mass spectrometry (MS) analyses comparing the protein composition of conditioned medium from poly (I:C) and mock-treated LL5 cells.

## 2. Material and Methods

### 2.1. Cell Growth

*L. longipalpis* embryonic LL5 cells obtained from Dr. Robert B. Tesh (University of Texas Medical Branch, Galveston, TX, USA) were grown at 30 °C in L15 medium (Sigma, Mendota Heights, MI, USA) supplemented with 10% fetal bovine serum (FBS; Hyclone, Logan, UT, USA), 10% Tryptose Phosphate Broth, and 1% antibiotics (penicillin 100 U/mL and streptomycin 100 mg/mL, Sigma).

### 2.2. Transfection and Conditioned Medium Collection

For mock transfection, 4 ng/mL Lipofectin Transfection Reagent (Invitrogen, Carlsbad, CA, USA) were added to L15 medium (Leibovitz) containing 20% Tryptose Phosphate Broth (TPB). The transfection mix contained the mock transfection mix added with 2 ng/mL of polyinosinic:polycytidilic acid (poly I:C), a synthetic analog of double-stranded RNA (Invitrogen). These mixtures were added to 8 × 10^7^ LL5 cells for 24 h and supernatant was aspirated and stored. New medium was added and supernatant was aspirated and stored after 24 h. For RNA preparation, transfected and mock-transfected cells were collected after 6, 12, 24, and 48 h incubations, as described above. Cell viability was verified by trypan blue staining. More than 98% of cells were viable in all experiments.

Protease Inhibitor Cocktail 1× (Sigma) was added to the media. Dead cells and large debris were pelleted by centrifugation at 2000× *g* for 10 min. Supernatants were centrifuged again at 10,000× *g* for 30 min to exclude remaining cell debris and microvesicles. The proteins in the supernatant were then precipitated using trichloroacetic acid (TCA).

### 2.3. Trichloroacetic Acid Precipitation

One volume of TCA 100% (Sigma) was added to four volumes of conditioned medium and incubated for 20 min at −20 °C. The samples were centrifuged at 10,000× *g* for 10 min. The supernatant was removed and the protein pellet was washed with cold acetone, vortexed and centrifuged at 10,000 × *g* for 5 min. The material was submitted to mass spectrometry.

### 2.4. Mass Spectrometry Analysis

The pellets were suspended in 6 M urea, 2 M thiourea, and 10 mM 4-(2-hydroxyethyl)-1-piperazineethanesulfonic acid (HEPES). The proteins were reduced with 1 mM Dithiothreitol (DTT) and 50 mM ammonium bicarbonate (ABC), and alkylated with 5.5 mM iodacetamide and 50 mM ABC. Before trypsinization, the samples were purified with detergent removal spin columns (Thermo Scientific). Then, the samples were digested in 50 mM ABC with trypsin (Promega, cat. V5113, Madison, WI, USA) in a 1:50 trypsin to protein mass ratio by incubation at 24 °C for 18 h. After trypsinization, trifluoroacetic acid (TFA) was added to a final concentration of 0.5%. Peptides were desalted with homemade C18 spin columns. The peptides were analyzed in triplicate by liquid chromatography tandem-mass spectrometry (LC-MS/MS) in a Thermo Scientific Easy-nLC 1000 system coupled to a LTQ Orbitrap XL ETD (mass spectrometry facility RPT02H/Carlos Chagas Institute–Fiocruz, Curitiba, PR, Brazil). Peptide separation was carried out in 15-cm (75-µm inner diameter) fused silica, in-house packed with reversed-phase ReproSil-Pur C18-AQ 3-µm resin (Dr. Maisch GmbH, Ammerbuch-Entringen). Chromatography runs were performed in a flow rate of 250 nL/min from 5 to 40% MeCN in 0.1% formic acid in a 120-min gradient, and a voltage of 2.3 kV was applied for peptide ionization. The mass spectrometer operated in a data-dependent acquisition mode. Survey full-scan MS spectra (at a 300–1650 *m*/*z* range) were acquired in the Orbitrap analyzer with resolution of 60,000 at *m*/*z* 400 (after accumulation to a target value of 500,000 in the C-trap). The ten most intense ions were sequentially isolated and fragmented in the linear ion trap using collision-induced dissociation at a target value of 30,000. The “lock mass” option was enabled at 445.120025 *m*/*z* in all full scans to improve mass accuracy of precursor ions [28]. Protein identification was performed with MaxQuant algorithm [29,30] version 1.4.1.2. Default parameters of the software were used for all analysis steps, unless stated otherwise. Proteins were searched against an *L. longipalpis* protein sequence database (containing 10,110 protein sequences from the VectorBase protein database, downloaded on 9 December 2013) and common contaminants, besides their respective reverse sequences to estimate the false discovery rate (FDR). Carbamidomethylation of cysteine was set as a fixed modification, while methionine oxidation and N-terminal acetylation (protein) were allowed as variable modifications. In addition, an FDR threshold of 0.01 was applied at both peptide and protein levels. Protein quantification was performed using a label-free approach, where the peptide peaks were detected as three-dimensional features—retention time versus signal intensity (extracted ion chromatogram, XIC) versus mass/charge—and were aligned and compared across the runs, as previously described [31].

### 2.5. In Silico Analyses

The amino acid sequences of identified secreted proteins were subjected to bioinformatic analyses. The protein signal peptide (SP) from 304 proteins was estimated using the software tool Prediction of Signal peptide “PredSi” at http://www.predisi.de./home.html [32], under standard configuration.

Protein–protein interactions were performed using the Search Tool for the Retrieval of Interacting Genes/Proteins (STRING) database V 10.0 at http://string.embl.de/ [33].

### 2.6. RNA Extraction and cDNA Synthesis

Three biological replicates of 8 × 10^7^ cells were transfected. The supernatant was removed 6, 12, 24, and 48 h post-transfection and the cells, after being washed three times with PBS, were resuspended in 1 mL of TRizol (Ambion, Waltham, MA, USA). The RNA was extracted following the TRizol manufacturer’s instructions. Total RNA was precipitated with isopropanol, resuspended in water and stored at −70 °C. RNA was treated with DNase I (Promega) and cDNA was synthesized from 5 μg of total RNA using SuperScript III First-Strand Synthesis (Invitrogen).

### 2.7. qPCR

Real-time polymerase chain reaction (qPCR) was performed using SYBR Green PCR Master Mix (Applied Biosystems, Foster City, CA, USA) and the primers listed in Table 1. Expression levels were determined through 2(-Delta Delta C(T)) method (DDCt) [34], normalized using *rp49* gene expression [35], yielding the relative expression value. Statistical analyses were done using GraphPad Prism software, version 5.04 (GraphPad Software, Inc., San Diego, CA, USA). For comparison of the transfected and mock samples at different times after transfection, an unpaired two-tailed Student’s *t* test was performed. * *p* ≤ 0.05, ** *p* ≤ 0.01, *** *p* ≤ 0.001.

### 2.8. Comparative Analysis of Transcript and Protein Levels

Comparative analysis of expression values was performed to verify the correlation between mRNA levels of LL5 cells (samples collected 6, 12, 24 and 48 h post transfection) evaluated by qPCR, and secreted protein levels (samples collected 24 or 48 h post transfection) evaluated by mass spectrometry. Linear regression, Pearson’s correlation coefficient (r), and goodness of fit (r^2^) were calculated using GraphPad Prism software (version 6.05).

## 3. Results and Discussion

Immune responses are triggered through the recognition of pathogen-associated molecular patterns (PAMPs) by host pattern recognition receptors (PRRs). Binding of PAMPs leads to the activation of signaling pathways: RNAi, Janus kinase/signal transducers and activators of transcription (JAK/STAT), Immune Deficiency (IMD) and Toll [36,37,38,39]. The major insect innate immune response triggered by virus infection is normally the small interfering RNA pathway. RNAi is a molecular mechanism in which the presence of intracellular dsRNA leads to the production of small RNAs that suppress the expression of genes with matching sequences [40]. Nevertheless, alternative mechanisms exist.

In previous work, our group identified an antiviral response when the LL5 embryonic *L. longipalpis* cell lineage was transfected with dsRNA, which included the synthetic poly I:C. After dsRNA transfection, cells did not support WNV virus like particle (VLP) replication, as evidenced by lack of luciferase reporter gene expression. This block in replication may be due to a cellular antiviral response triggered by dsRNA. [22]. A similar non-specific antiviral response was observed in the marine shrimps *Litopanaeus vannamei* and *Penaeus monodon* [41,42]. More recently, the occurrence of dsRNA-mediated non-specific antiviral response was also described in two members of the Apidae family: the honey bee *Apis melifera*, and the bumblebee *Bombus terrestris* [43,44,45]. This non-specific response differs from what is seen in *Drosophila*, where transfection with virus-specific dsRNA triggers an RNA interference (RNAi)-mediated response against the virus, whereas non-related dsRNAs fail to activate the antiviral response [46].

In vertebrates, intracellular dsRNA is recognized by the PRR retinoic acid-inducible gene 1 (*RIG-I*) and the melanoma differentiation-associated protein (MDA5). These are also known as RIG-I-like receptors (RLRs) [47,48]. These receptors recruit the mitochondrial antiviral-signaling (MAVS) protein that mobilizes kinases and ubiquitin ligases, leading to the activation of transcription factors, such as interferon receptor factor 3 (IRF-3) and nuclear factor κB (NF-κB) [49,50]. The transcription factors induce type I interferon (IFN) and pro-inflammatory cytokine production. dsRNA, including poly I:C, is also detected by Toll-like receptor 3, initiating the type-I interferon (IFN-α, β) signaling pathway via a Toll/interleukin-1 receptor (TIR)-domain-containing adapter-inducing interferon-β (TRIF) signal, which activates interferon receptor factor 3 (IRF-3) and nuclear factor κB (NF-κB), leading to IFN-β expression [51,52].

To investigate variations in the exoproteome profile of dsRNA-transfected *L. longipalpis* LL5 cells, we carried out mass spectrometry (MS) analyses comparing the protein composition of conditioned medium from poly I:C and mock-treated cell cultures at two time points (24 and 48 h); in total 304 proteins were identified (Table S1).

Only 17.6% (53) of all identified secreted proteins presented a signal peptide (SP). Among proteins with ≥2-fold positive change variation, this percentage decreased to 11.53%, with only six proteins with SP: 2 at 24 h and 4 at 48 h (Table S1). The lack of signal peptide in the majority of secreted proteins suggests that unconventional protein secretion pathways are the main secretory mechanisms in LL5 cells, as already seen in other systems [53]. This could be due at least partly to exosomal transport. We identified some of the known exosome markers among our secreted proteins, based on the Exo Carta exosome marker list (www.exocarta.org/exosome_markers). Among these were glyceraldehyde-3-phosphate dehydrogenase (GAPDH), enolase (ENO), fructose-bisphosphate aldolase (ALDOA), moesin (MSN), phosphoglycerate kinase (PGK), cluster of differentiation (CDs) 37, 48 and 63, the heat shock proteins (HSPs) 70 and 90, and annexins (Table S1). These were not differentially expressed with a greater than 2-fold change and were present in both mock and poly I:C transfection samples.

**Figure 1:**
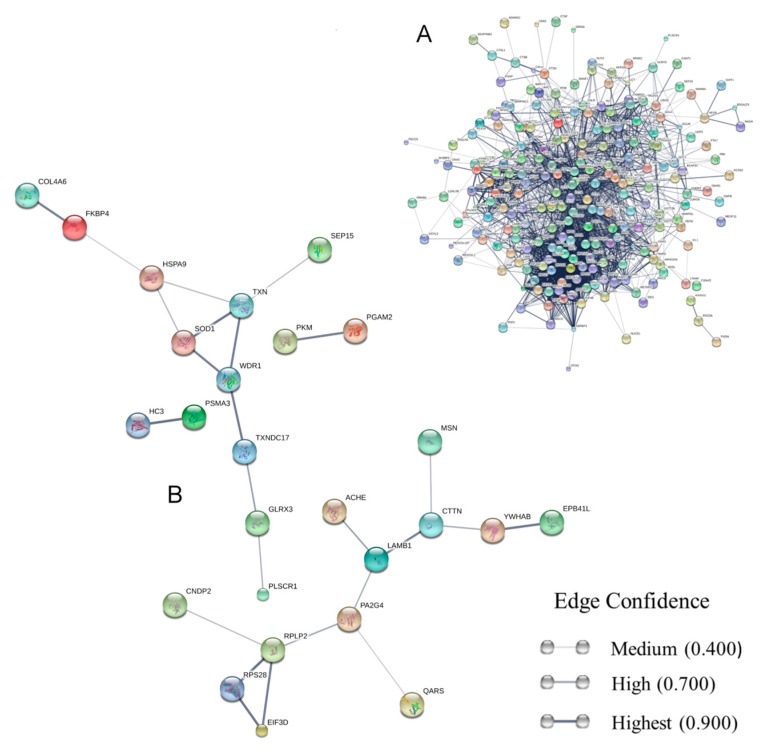
Secretome networks showing predicted functional linkages between identified proteins. (**A**) Interaction map of the whole LL5 double-stranded RNA (dsRNA)-transfected cell exoproteome; (**B**) interactome map of the secreted proteins with positive variation of ≥2-fold change. The STRING interactome maps were generated using default settings (medium confidence of 0.4, with 7 criteria for linkage: neighborhood, genefusion, co-occurrence, co-expression, experimental evidence, existing databases, and textmining). Proteins were represented as nodes and their functional links were defined by solid lines. The thickness of the lines signifies the level of confidence of the reported association. The non-connected nodes were suppressed. No additional interplay proteins were added to the networks. The protein symbols and node colors are listed in Table S2.

We performed an interaction analysis of the LL5 dsRNA-transfected exoproteome. The network analysis of 304 proteins presenting different abundance between mock and transfected groups (Table S1) predicted that 271 proteins (Table S2) were connected by 2184 edges (Figure 1A) forming an intricate network. The prevised protein interaction (PPI) enrichment *p*-value was 0.0, meaning that secreted proteins have more relations among themselves than what would be expected for a random set of proteins of similar size drawn from the genome. Exoproteome functional enrichment analysis identified enhanced interactions related to specific biological functions. These interactions have a significant false discovery rate (FDR) of less than 0.05. Among these gene ontology (GO) categories and pathways, we can quote: biological process GO:0006518 (peptide metabolic process) FDR: 1.42 × 10^−24^; cellular component GO:0070062 (extracellular exosome) FDR: 4.43 × 10^−70^; molecular function GO:0003733 (RNA binding) FDR: 3.45 × 10^−22^; and the Kyoto Encyclopedia of Gene and Genomes (KEGG) pathway ID:03010 (Ribosome) FDR: 8.4 ×10^−15^.

**Figure 2:**
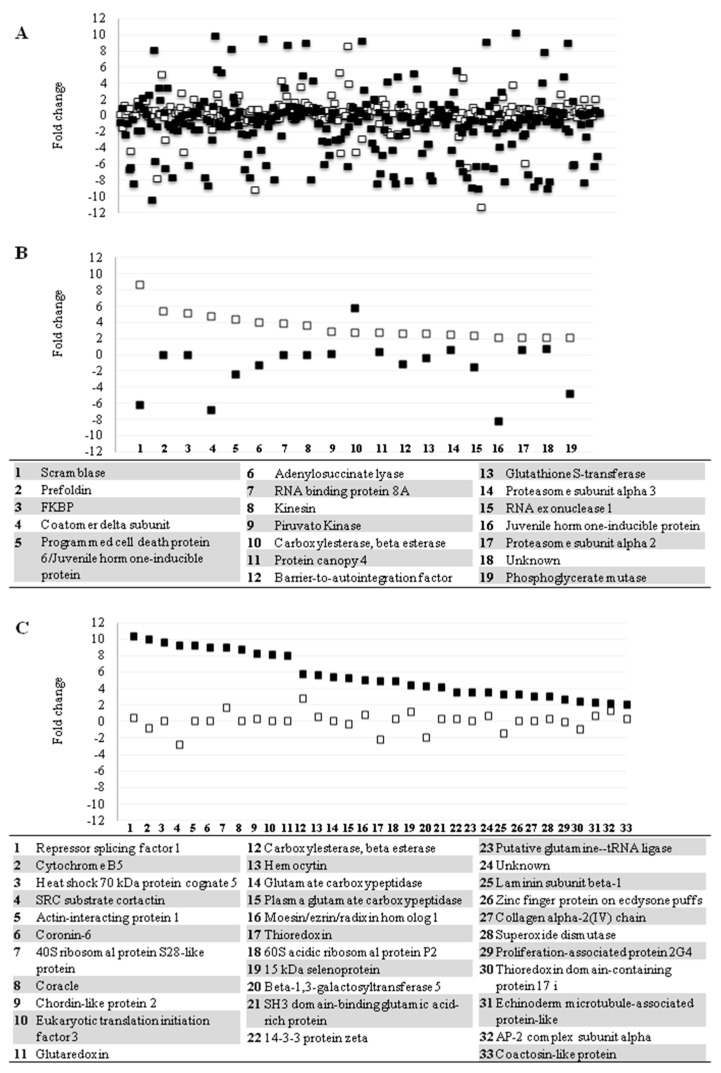
LL5 cells protein secretion behavior in response to dsRNA treatment. (**A**) Variation of relative protein abundance in the whole exoproteome of LL5-transfected cells at 24 and 48 h post-transfection; (**B**) secretion profile behavior of proteins with amount variation of ≥2-fold change at 24-h; (**C**) secretion profile behavior of proteins with amount variation of ≥2-fold change at 48-h. White squares represent the proteins’ relative amount variation at 24-h after transfection. Black squares represent the proteins’ relative amount variation at 48-h after transfection. Squares in the same point of the X-axis represent the same protein. Numbers below graphs (**B**,**C**) represent proteins listed in corresponding inset.

We also carried out STRING interaction analysis (Figure 1B) of proteins with ≥2-fold change variation detected on mass spectrometry analysis. From the 51 protein sequences (Table S2) shown in Figure 2B,C, 45 were identified in the STRING database (Table S2) and from those, 26 (Table S2) (Figure 1B) were predicted as connected at least to one protein. This interaction analysis displayed a significant PPI enrichment *p*-value of 4.51 × 10^−4^. In this interactome we can distinguish two major protein groups: the first one with 12 members is related to oxidative stress modulation and includes phospholipid scramblase (PLSCR1), thioredoxin (TXN), glutaredoxin (GLRX3), superoxide dismutase (SOD1), and forskolin-binding protein 4 (FKBP4), among others. The second group is comprised of 10 proteins linked with peptide synthesis and cytoskeleton organization. Among these are the riboproteins RPS28 and RPLP2, the eukaryotic translation initiation factor 3 (EIF3D), moesin (MSN), cortactin protein (CTTN), and others. Two biological functions were enhanced in the ≥2-fold change secreted protein cluster: cellular component: GO: 0070062 (extracellular exosome) FDR: 5.58 × 10^−12^ and molecular function: GO.0003723 (RNA binding) FDR: 4.41 × 10^−2^. These same biological function enrichments are found when we study the whole exoproteome. The complete FDR-filtered (*p* < 0.05) lists are shown in Table S3.

The measurement of relative protein abundance revealed that the secretion of most proteins did not vary significantly between test and control supernatants (Figure 2A). Only 6.95% (18) of all identified proteins at 24 h presented variation of ≥2-fold change, and 6.22% (17) presented variation of ≤2-fold change (Figure 2B). These percentages change to 11.54% (33) and 24.83% (71), respectively, at 48 h after dsRNA transfection (Figure 2C). We focused on the 51 proteins with ≥2-fold change variation, which represent 17.28% of the total identified proteins (Figure 2C).

The proteins with intensified secretion in the first 24 h had their relative amounts decreased at 48 h. Most proteins more abundant at 48 h had a basal detection level at 24 h. Interestingly only one protein, the enzyme carboxylesterase, presented secretion levels with ≥2-fold change at both time points. In insects this enzyme is related to detoxifying processes, including insecticide resistance [54]. Several proteins with higher secretion at 24 h or 48 h are related to immune responses. Among the proteins with higher secretion at 24 h we can highlight eight proteins related to immune response. Phospholipid scramblase 1 (PLSCR1) had a positive variation with a greater than 8-fold change. Scramblases are conserved in all eukaryotic organisms. They are multiple palmitoylated, endofacial membrane proteins originally identified based on their capacity to promote accelerated transbilayer phospholipid movement in response to Ca^2+^ [55]. PLSCR1 was identified as an interferon and growth factor-inducible protein necessary for the IFN-dependent induction of gene expression and antiviral activity [56]. In human and mouse plasmacytoid dendritic cells (pDCs), which are professional interferon-producing cells specialized in recognizing viral RNA and DNA through the endosomal Toll-like receptors (TLRs) TLR7 and TLR9, respectively, PLSCR1 was described as a TLR9-binding protein that plays a significant role in type-1 interferon responses in pDCs by regulating TLR9 expression and trafficking [57]. The direct interaction of PLSCR1 with human T-cell leukemia virus type-1 (HTLV-1) leads to a reduction of virus titers [58]. These reports demonstrate the significant, but not fully understood, role of phospholipid scramblase in antiviral response.

Another interesting protein with a greater than 5-fold increased secretion at 24 h was the hexameric chaperone complex prefoldin (PFD). Subunits of prefoldin have been found to be associated with cellular response to viral infection. The PFDN1 expression was positively modulated in both vaccinia virus-infected HEK 293 cells and in the RABV-infected N2a cells [59,60]. PFDN2 was found directly bound to the hepatitis C virus (HCV) F protein [61].

Still within the proteins with higher secretion at 24 h, we found forskolin-binding protein 59 (FKBP59), an immunophilin family member, with a secretion level increase of greater than 5-fold. This is a highly conserved group of proteins which binds to immunosuppressive drugs such as FK506, rapamycin, and cyclosporine [62,63]. FKBPs are involved in several biochemical processes and participate in the immune response against viral infection. Mitochondrial FKBP51 was identified as a tumor necrosis factor (TNF) receptor-associated factor 3 (TRAF3) and TNF receptor-associated factor 6 (TRAF6) binding protein. Binding of FKBP51 to TRAF proteins facilitates the type-I interferon response induced by dsRNA transfection or Newcastle disease virus (NDV) infection in murine fibroblasts. Depletion of FKBP51 reduced the expression of type 1 INF in these cells [64].

One interesting protein with a greater than 5-fold enhanced secretion at 24 h is coatomer protein I (COPI). COPI-coated vesicles are associated with transport from the Golgi to the endoplasmic reticulum [65], endocytosis [66], maturation of autophagic vacuoles [67], expression of cell surface receptors, and modulation of the plasma membrane lipid composition [68]. Studies demonstrated the involvement of COPI subunits in diverse aspects of virus infection [69,70,71]. Depletion of the COPI β 2 subunit in human 293/ACE2 cells led to a decrease in the severe acute respiratory syndrome–coronavirus (SARS-CoV) replication [72].

We identified two juvenile hormone-inducible proteins at 24 h with 4.43- and 2.12-fold increases. Despite the absence of information on the possible involvement of juvenile hormone-inducible proteins in the insect antiviral response, the juvenile hormone itself and retinoic acid are terpenoids, with similar structure and biological effects in mammalian and insect cells [73]. Retinoic acid-inducible genes are related to type-I IFN response in mammals [48,74].

Another virus infection-related protein found was kinesin, an ATPase protein belonging to the class of motor proteins. Kinesins move along microtubule (MT) filaments, and are power-driven by the hydrolysis of adenosine triphosphate (ATP) [75]. Recently this protein has been associated with the intracellular transport of different viruses. HIV-1 trafficking to the nucleus is kinesin- and dynein motor-dependent [76]. Kinesin was also identified as a Chandipura virus matrix binding protein [77]. Kinesin from lepidopteran *Trichoplusia ni* binds directly to nucleocapsid proteins of the *Autographa californica* multiple nucleopolyhedrovirus (AcMNPV) [78], and the α herpersviral envelope protein pUS9 uses kinesin 1 to promote the anterograde axonal transport of herpes simplex virus 1 (HSV-1) [79]. These results demonstrate that kinesin protein interacts directly with the virus particle.

The insect protein canopy 4 is a homolog of the human protein associated with Toll-like receptor (TLR) 4 (PRAT4). PRAT4 is required for cell surface expression of the Toll family of receptors and for the intracellular nucleic acid-sensing TLR7/9. This protein is essential for TLR-dependent immune response [80]. The 2.7-fold increased secretion of this PRAT4 homolog signals the participation of Toll-like receptors in the LL5 antiviral non-specific immune response.

The barrier to autointegration factor (BAF) DNA binding protein had an increase of 2.65-fold at 24 h. This protein is described as a potent antiviral effector against poxyviruses, retroviruses and the herpes virus. The BAF antiviral property is regulated by kinases and phosphatases, with dephosphorylation activating its antiviral properties [81].

Notwithstanding the higher number of positively modulated secreted proteins at 48 h when compared with 24 h, the number of proteins described in immune response to viruses in interferon-like responses was smaller than what was seen at 24 h. Among them we quote the protein hemocytin, with a secretion increase of 5.66-fold. Hemocytin is an insect humoral lectin homologous to mammalian von Willebrand factor, which is involved in platelet adhesion to subendothelium [82]. Hemocytin expression is induced by pathogens. Lectins are adhesive molecules which bind carbohydrates and among other functions are involved in hemolymph bacterial clearance [83]. Also of interest is the occurrence of glutaredoxin (fold change of 7.96), thioredoxin (fold change of 4.95) and superoxide dismutase (fold change of 2.99), antioxidant enzymes involved in the control of oxidative stress at 48 h. Reactive oxygen species (ROS) production, generated due to microbial invasion, has long been known to exert an antimicrobial effect. The activation of the antiviral and inflammatory signaling pathways has also been linked with the production of ROS [84,85]. Due to the high chemical reactivity of ROS, cells possess scavenger antioxidant mechanisms that maintain redox homeostasis [86].

**Figure 3:**
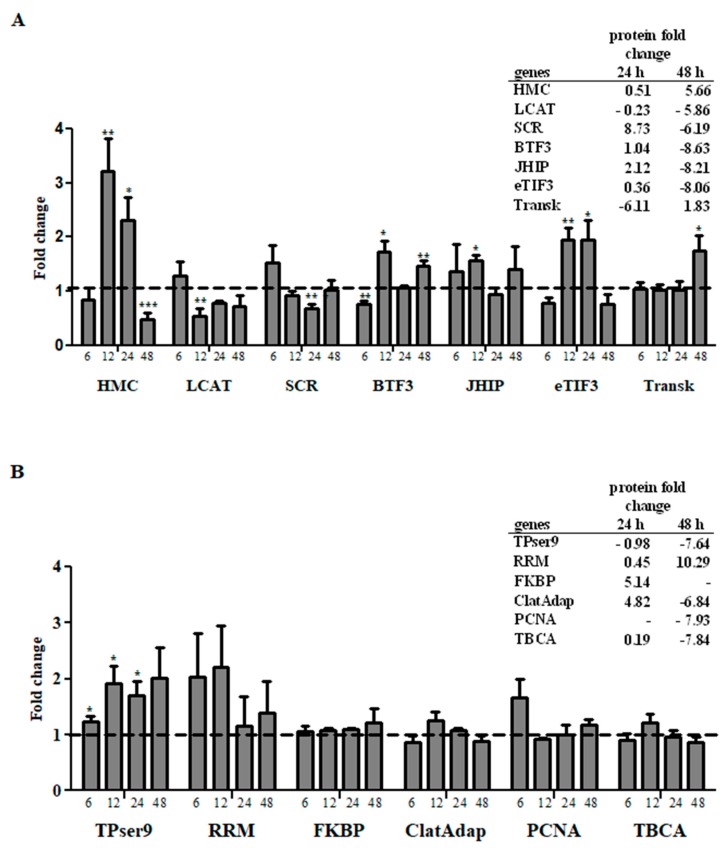
Transcriptional profile of genes involved in antiviral response. (**A**) Genes with correlated transcriptional and protein secretion profiles on mass spectrometry at 24 h or 48 h post-transfection; (**B**) genes with non-correlated transcriptional and protein secretion profiles on mass spectrometry on mass spectrometry at 24 h or 48 h post-transfection. Y-axis represents fold change in gene expression relative to the mock-transfected group (horizontal traced line) normalized to the *rp49* reference gene. The X-axis represents gene name (Table 1) and samples collected at different time points post-transfection. Quantification was normalized relative to the house keeping gene *rp49*, and relative gene expression expressed as fold change was calculated relative to the mock group. Bars represent mean with standard error (SEM) of two biological replicates. Tables represent protein fold change corresponding to each gene name. * *p* ≤ 0.05, ** *p* ≤ 0.01, *** *p* ≤ 0.001.

Real time PCR (qPCR) analysis of 13 genes coding for significantly positive- or negatively-modulated secreted proteins revealed the existence of some genes with transcriptional profile correlated with the amount of protein detected in the supernatants (Figure 3A). There were seven molecules that presented a correlation between mRNA and secreted protein levels (represented in Figure 3A). Three of them, hemocytin (HMC), lecithin cholesterol acyltransferase (LCAT), and scramblase (SCR), showed coordinated mRNA and secreted protein levels. When mRNA levels changed at 12 or 24 h post transfection (either by up- or down-regulation), we detected a corresponding change in secreted protein levels 12 or 24 h after the mRNA level changes. Hemocytin (HMC), an insect humoral lectin which plays an important role in a non-specific self-defense mechanism [82], had increased mRNA and secreted protein levels at 12 h and 24 h. This indicates that the transcription, translation and secretion of this molecule are correlated and the prolonged increase of its expression suggests this molecule may be important for the LL5 cells response to poly I:C transfection.

Two other molecules, lecithin cholesterol acyltransferase (LCAT) (a plasma enzyme which esterifies cholesterol) [87], and scramblase (SCR) (a regulator of transbilayer lipid asymmetry in eukaryotic cell membranes) [55], were reduced both in mRNA and secreted protein levels at 12 or 24 h post-transfection. These two molecules are involved in lipid metabolism or membrane formation and the reduction of their expression may suggest that LL5 cells reduce vesicle formation 48 h after transfection challenge.

Three other molecules showed increased mRNA levels at 12 or 24 h post transfection and increased secreted protein levels at 24 h, with posterior decrease at 48 h. One was the eukaryotic translation initiation factor 3 (eTIF3), which in humans is directly recruited by the hepatitis C virus (HCV) internal ribosome entry site (IRES) to promote the translation of viral proteins [88]. In LL5 cells it showed increased mRNA levels at 12 h and 24 h, and increased secreted protein levels 24 h after mRNA increase. Curiously, 48 h post transfection the secreted protein levels decreased drastically. Juvenile hormone-inducible protein (JHIP) showed increased mRNA levels after 12 h and increased secreted protein levels 24 h after mRNA increase. Curiously, at 48 h post transfection the secreted protein levels decreased drastically regardless of changes in mRNA levels prior to this timepoint. Basal transcription factor 3 (BTF3) has been reported to play a significant role in the transcriptional regulation related to eukaryote growth and development [89]. In LL5 cells it showed increased mRNA levels at 12 h and increased secreted protein level 12 h after mRNA increase. At 48 h post transfection the secreted protein levels decreased drastically regardless of changes in mRNA levels prior to this time point.

Transketolase mRNA levels increased at 48 h post transfection and the secreted protein levels also increased at this same time point, suggesting a late effect. Curiously, regardless of the maintenance of mRNA levels from 6 h to 24 h, the secreted protein levels were considerably low at 24 h post transfection. Changes in transketolase activity are involved in a number of tumors and degenerative diseases [90].

Based on the data presented in Figure 3A, we observed that in the majority of the molecules (six out of seven) there was a correlated change of secreted protein levels 12 h to 24 h after changes in mRNA levels. The remaining genes investigated showed no correlation between the mRNA levels and the detection of proteins in the supernatants. (Figure 3B).

**Figure 4:**
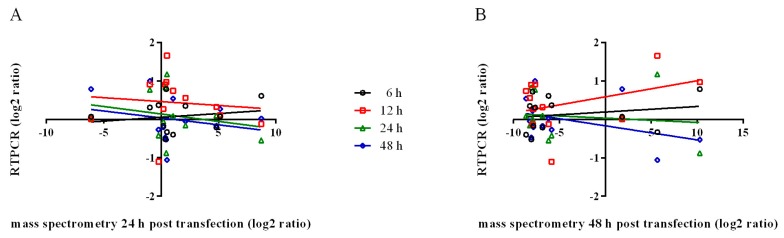
Comparison of transcript and secreted protein abundance. Mass spectrometry expression values (log2 ratio) of LL5 cell-secreted proteins collected at 24 h (**A**) or 48 h (**B**) post transfection were plotted on X-axis; mRNA levels obtained from qPCR (log2 ratio) of LL5 cells collected at 6 h (black circles), 12 h (red squares), 24 h (green triangles), and 48 h (blue diamonds) post-transfection were plotted on the Y-axis. Lines represent linear regression.

The linear regression of mRNA levels plotted against secreted protein levels revealed a weak correlation (Figure 4). The Pearson correlation coefficient (r) and goodness of fit (r^2^) calculated for mRNA levels (6 h, 12 h, 24 h and 48 h) versus secreted protein (24 h and 48 h) are shown in Figure 4 and Table 2. The non-correlation between transcription levels and amounts of secreted protein indicates that secretion is regulated by some not-yet disclosed mechanism, involving transcription, translation, storage, sorting, and transport, as already reported for other systems [91]. This non-correlation was also observed when the exoproteome of WRL-68 human hepatic cells infected with the chikungunya virus was analyzed [92].

We observed that the majority of analyzed genes were transcribed in the first 24 h (Figure 3A,B). The results obtained with the signal peptide prediction, the secreted protein interaction network, the identified biological functions enriched pathways, and the relatively small number of proteins with significant abundance variation reveal the existence of finely-tuned protein secretion modulation in response to dsRNA treatment. There is also a strong indication for the participation of the exosomal secretory pathway as the major LL5 cell secretion mechanism in this response. The increased secretion of proteins such as phospholipid scramblase, FKBP, juvenile hormone-inducible proteins, and protein canopy 4 in the first 24 h indicate that this non-specific antiviral response is in a way similar to an interferon-like response mediated by the Toll pathway. This response occurs preferentially in the first hours after transfection. The increase of antioxidant enzymes at 48 h indicates that the cells are working to control the oxidative stress level in the culture.

We are presently in the process of validating these findings by silencing specific genes with subsequent analysis of nonspecific antiviral response.

## Figures and Tables

**Table 1:** List of primers used for qPCR.

Gene	Primer Name	Sequences
Hemocytin	HMC-F	5′AAGGATGCCTGCAATAGGTG3′
	HMC-R	5′CCTCCGGAACACAGACAAAT3′
Lecithin-cholesterol acyltransferase	LCAT-F	5′AGCCGTGAAGGTCTTTGCCATT3′
	LCAT-R	5′TGCCAGGGATGGGGATGTAATTTG3′
Scramblase	SCR-F	5′CCATGCGCCCATTTGACATGAA3′
	SCR-R	5′GATTGACAGCGCAATGGACGATAG3′
Basic transcription factor 3	BTF3-F	5′AGTGCATAAGCAGGCAACACCA3′
	BTF3-R	5′TACGTCGCCACCGAAATGAGTT3′
Juvenile hormone-inducible protein	JHIP-F	5′CCTTGCTGAGCTCCTTGAGAAACT3′
	JHIP-R	5′TACACATGGCCATTCCCATCTTCC3′
Eukaryotic translation initiation factor 3	eTIF3-F	5′TCGATAGGCATCTCACGTTTCCAC3′
	eTIF3-R	5′TCTTCCCCACTGTATCCAGGATGT3′
Transmembrane protease serine 9-like	TPser9-F	5′TCCACAATCCGGATGCAGACATAG3′
	TPser9-R	5′CCAACCCGATTGACGATCTCAGAA3′
Transketolase	Transk-F	5′TGTTAGCTGCGAACGTGCTGTA3′
	Transk-R	5′GTATTTGGTCGGGATGTGCGAATG3′
Repressor splicing factor	RRM-F	5′AATTTGGGAAGCTCAATA3′
	RRM-R	5′GAGGATGTCGCAAGCCTTCT3′
forskolin-binding protein	FKBP-F	5′TGAGTTTGAACGTGCCCAGGAT3′
	FKBP-R	5′CTCCTTGATGTACTTGGCACCCTT3′
Coatomer delta subunit	ClatAdp-F	5′TAGCCGATGAGAAGTTCGGGAAGA3′
	ClatAdp-R	5′CTTATCGACATTGGGGTGCGTTTG3′
Proliferating cell nuclear antigen	PCNA-F	5′CATGAATCTCGACCAGGAGCACTT3′
	PCNA-R	5′TCACGGCAAATGCGTGCAAA3′
Tubulin-specific chaperone A	TBCA-F	5′CGTACGAAAAGGAAGCAGATCAGCA3′
	TBCA-R	5′CTTCCTTCCGGATCACGTGTTCAT3′
Ribosomal protein 49	rp49-F	5′GACCGATATGCCAAGCTAAAGCA3′
	rp49-R	5′GGGGAGCATGTGGCGTGTCTT3′

**Table 2:** Pearson correlation coefficient (r) and goodness of fit (r^2^) calculated for expression values from mRNA levels against secreted protein levels.

Samples	6 h mRNA		12 h mRNA		24 h mRNA		48 h mRNA	
	r	r^2^	r	r^2^	r	r^2^	r	r^2^
**24 h secreted protein**	0.1742	0.0304	−0.1070	0.0115	−0.2399	0.0576	−0.2265	0.0513
**48 h secreted protein**	0.2024	0.0410	0.3754	0.1409	−0.1197	0.0143	−0.4018	0.1615

## Supplementary Materials

The following are available online at www.mdpi.com/1999-4915/10/1/43/s1. Table S1: Identified proteins; Table S2: Interactome; Table S3: String GO Tables.
